# Tables of Diagnostic Pupillary Drug Tests

**Published:** 1975-04

**Authors:** H. Stanley Thompson

**Affiliations:** Iowa City, Iowa, U.S.A.


### Tables of Diagnostic Pupillary Drug Tests

By

**H. Stanley Thompson, M.D.,**

Iowa City, Iowa, U.S.A.

There are both sympathetic and parasympathetic nerves to the iris muscles. When the innervation of one iris is disturbed, an anisocoria becomes apparent and very distinct and characteristic changes take place in the responsiveness of the denervated muscle to drugs depending on the location and the completeness of the denervation.

Thus it becomes possible to localize parasympathetic lesions by watching the changes in pupil size in response to cholinergically active drugs (i.e. miotics); and sympathetic lesions can be identified by the response of the pupil to adrenergically active drugs (mydriatics).

These two tables are designed for ready reference —as a practical guide to diagnostic pupillary drug tests. The tables will not tell you why these drugs behave as they do. The reader who wants to know more about the anatomy and pharmacology on which these tests are based could start with the further reading listed below.

**Some Cautionary Notes:**

With most drugs use two drops, one minute apart just in case most of the first drop is washed away.

Look at the pupils 30 minutes after cholinergic drugs, and 45 minutes after adrenergic drugs.

Ideally, photos should be taken before and after, but if this is not possible, at least measure the pupils and write down their diameters before and after.

There are large interindividual differences in the responsiveness of the iris to topical drugs. This becomes most evident when weak concentrations are used. For example, 0.25% pilocarpine will produce a minimal construction in some patients and an intense miosis in others. This means that the most secure clinical judgements stem from comparisons with the action of the drug on the other, normal eye. It should also be remembered that the general status of the patient will influence the size of the pupils. If the patient becomes uncomfortable or anxious while waiting for the drug to act, then both pupils may dilate. If the patient becomes drowsy, both pupils will constrict. So, if a judgement is to be made about the dilation or contraction of the pupil in response to a drug placed in the conjunctival sac, **one pupil should be used as a control whenever possible.** If only one eye is involved, the drug should be put in both eyes so that the response of the normal and abnormal eye can be compared. When the condition is bilateral no such comparisons are possible, but an attempt should be made to make sure that the observed response is indeed caused by the instilled drug. In such a case the drop should be put in one eye only so that the responses of the medicated and unmedicated eyes can be compared.

The weaker concentrations of pilocarpine are an adequate substitute for methacholine 2.5%.

Cocaine and hydroxyamphetamine should be used

Table 1

PARASYMPATHETIC DEFECT

| Topically applied Drug | Normal | Third nerve lesion e.g. aneurysm (preganglionic) | "Adies" tonic pupil (postganglionic) | Atropinized pupil (pharmacologic blockade) |
|---|---|---|---|---|
| Mecholyl 2.5% | 0<br>No miosis | 0 → +<br>No miosis | 0 → + → +++<br>Mild-marked miosis | 0<br>No miosis |
| Pilocarpine 0.125% | 0 → +<br>Minimal miosis | +<br>Minimal miosis | ++ → +++<br>Moderate→marked miosis | 0<br>No miosis |
| Pilocarpine 0.25% | +<br>Mild miosis | + → ++<br>Mild miosis | +++ → ++++<br>Marked miosis | 0<br>No miosis |
| Pilocarpine 0.50% | ++<br>Moderate miosis | ++<br>Moderate miosis | ++++<br>Intense miosis | 0<br>No miosis |
| Pilocarpine 1.0% | +++<br>Marked miosis | +++<br>Marked miosis | ++++<br>Intense miosis | 0<br>No miosis |

Table 2

| Drug | Normal | Central | Preganglionic | Postganglionic |
|---|---|---|---|---|
| | | SYMPATHETIC DEFECT | | |
| Cocaine 2%—10% (2 drops) | Mydriasis | Impaired dilation | No dilation* | No dilation* |
| Hydroxyamphetamine 1% (2 drops) | Mydriasis | Normal dilation; pupils become equal | At least normal dilation; Horner's pupil may become slightly larger | No dilation* |
| **Supersensitivity Tests** | | **Least dilation** | **Some dilation** | **Most dilation** |
| a) Epinephrine 1 : 1000 (several drops) | No dilation | No dilation | Moderate dilation | Dramatic dilation |
| b) Phenylephrine 10% (1 drop, viscous) | Dramatic dilation | Normal dilation; pupils become equal | At least normal dilation; Horner's pupil may become slightly larger | Horner's pupil dilates sooner, faster and more than normal; becomes noticeably larger |
| c) Phenylephrine 2% (2 drops) | Slight dilation | Slight dilation | Slight to moderate dilation | Moderate to dramatic dilation |

*Partial dilation suggests a partial defect on separate days since it is likely that each interferes with the other's mydriatic action.

The supersensitivity tests for Horner's syndrome should be avoided, for the following reasons: 1) a slightly increased dilation of the Horner's pupil does not distinguish a pre- from a postganglionic lesion with certainty; 2) these tests are based on the assumption that the same amount of the drug has reached each iris. After an ophthalmic examination, this may not be the case, since more of the drug may get through one cornea than through the other. **Supersensitivity tests are not valid unless done through untouched corneas.** 3) These tests have now been replaced for clinical purposes by the hydroxyamphetamine test.

If you have a patient with a ptosis and miosis, cocaine will help you to be sure that you are dealing with a Horner's syndrome—by failing to dilate the miotic pupil. But the cocaine test is not much help for localizing the lesion: any Horner's pupil dilates poorly with cocaine. This is to be expected from the mode of action of cocaine.

Hydroxyamphetamine (Paredrine, SKF) is a mydriatic drug only because it releases norepinephrine from the sympathetic nerve endings in the dilator muscle. Therefore it will fail to act only when there is a postganglionic lesion causing the Horner's syndrome.

Notice that there is no pupillary drug test which clearly distinguishes central from preganglionic lesions.

### REFERENCES

1) THOMPSON, H. S., Newsome, D., Loewenfeld, I. E.: The fixed dilated pupil: Sudden iridoplegia or mydriatic drops? A simple diagnostic test. Arch Ophthalmol **86**: 21-27 (1971).
2) THOMPSON, H. S. & Mensher, J. H.: Adrenergic mydriasis in Horner's syndrome. Am J Ophthalmol **72**: 472-480 (1971).
3) THOMPSON, H. S.: Diagnostic pupillary drug tests. In **Current Concepts in Ophthalmology,** Chapter 6, Vol. III, FC Blodi (ed.), CV Mosby Co., St. Louis (1972).
4) THOMPSON, H. S.: Medikamentöse Pupillendiagnostik. Die Normale und die gestörte Pupillenbewegung. **D.O.G. Bad Nauheim Symposium.** 1972. Dodt, E. und Schrader, K. E. (eds.) Munchen, J. F., Bergman (1973).

